# NaHSO_4_-SiO_2_ as an efficient and chemoselective catalyst, for the synthesis of acylal from aldehydes under, solvent-free conditions

**DOI:** 10.1186/1752-153X-6-136

**Published:** 2012-11-13

**Authors:** Ravi Kumar Kannasani, V V Satyanarayana Peruri, Srinivasa Reddy Battula

**Affiliations:** 1 RA Chem Pharma Limited, Research and Development Division, Prasanth Nagar, Hyderabad, AP, India; 2 Department of Chemistry, Acharya Nagarjuna University, Nagarjuna Nagar, Guntur, AP, India

**Keywords:** Acylals, Aldehydes, Solvent-free conditions, Reusable catalyst, NaHSO_4_-SiO_2_

## Abstract

**Background:**

Structurally diverse aldehydes are successfully converted into acylals (1,1-diacetates) with acetic anhydride using NaHSO_4_-SiO_2_ as a mild, convenient and inexpensive catalyst under solvent-free conditions. The noteworthy features of the present system are shorter reaction times, and mild and solvent-free conditions. Furthermore, it offers chemoselective protection of aldehydes.

**Results:**

Both aromatic and aliphatic aldehydes reacts smoothly with acetic anhydride in presence of silica supported sodium hydrogen sulphate to afford the corresponding 1,1-diacetates in good to excellent yields. We studied competitive reactions for the acylation of aldehydes in the presence of ketones using silica supported sodium hydrogen sulphate as a catalyst. Using this catalytic system, the highly selective conversion of an aldehyde in the presence of ketone was observed.

**Conclusions:**

NaHSO_4_-SiO_2_ is a chemoselective and highly efficient catalyst for acylal formation from aldehydes. The advantages of this methodology over the reported methods is the availability of the starting materials, simplicity of acylation procedure, a clean work-up, a short reaction time, high yields and reusability.

## Introduction

The concept of green chemistry has been playing an important role in recent years for meeting the fundamental scientific challenges of protecting the living environment. One of the thrust areas for achieving this target is to explore alternative reaction conditions and reaction media to accomplish the desired chemical transformation with almost negligible by products and waste generation as well as elimination of the use of volatile and toxic organic solvents. It is therefore of utmost importance to evolve a simple and effective methodology for the different organic transformations that cover the concept of green chemistry.

During the multi-step synthesis, the protection of the carbonyl group is widely achieved by the formation of acylal (1,1-diacetate) [1]. These substrates are important because of their application as precursors for the synthesis of 1-acetoxy dienes, valuable synthetic intermediates for Diels-Alder cycloaddition reactions [2]. The relative acid stability of acylal is another interesting feature in the field of protection and deprotection chemistry. General, acylals are prepared by treating aldehydes with acetic anhydride in the presence of protonic acids [3], Lewis acids [4], heteropoly acids, or clays [5]. Some examples of the reagents and catalysts that have been developed for this purpose include LiOTf [6], ceric ammonium nitrate [7], InCl_3_[8], H_2_NSO_3_H [9], LiBF_4_[10], H_2_SO_4_[11], PCl_3_[12], NBS [13], I_2_[14], TMSCl-NaI [15], FeCl_3_[16], Fe_2_(SO_4_)_3_.xH_2_O [17], Zn(BF_4_)_2_[18], Cu(BF_4_)_2_.xH_2_O [19], H_2_SO_4_-SiO_2_[20], Silica sulphate [21]. Although some of these methods have convenient protocols with good to high yields, the majority of these methods suffer at least from one of the following disadvantages: reaction under oxidizing conditions, prolonged reaction time, high temperatures, use of moisture-sensitive and expensive catalysts, use of solvents, strong conditions, difficulty in scaling up, etc. Therefore, development of catalysts working under mild reaction conditions is desirable.

In recent years, Heterogeneous catalysts have gained importance in several organic transformations due to their interesting reactivity as well as for economic and environmental reasons. In continuation of our research work to develop new methodologies for organic transformations, [22-25] we observed that silica supported sodium hydrogen sulphate is a highly efficient catalyst for the synthesis of 1,1-diacetates (acylals) from the reaction of aldehydes with acetic anhydride under solvent-free conditions. The catalyst NaHSO_4_-SiO_2_ can easily be prepared [26] from the readily available NaHSO_4_ and silica gel (230–400 mesh) and these are inexpensive and nontoxic as the reaction is heterogeneous in nature, so the catalyst can easily removed by simple filtration.

## Results and discussions

**Scheme 1:**
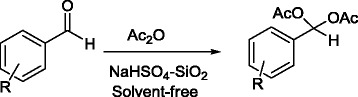
**Synthesis of 1**,**1-diacetates from aldehydes.**

Herein we wish to report an extremely convenient, mild, and highly chemoselective procedure for the conversion of aldehydes to the corresponding acylals in the presence of acetic anhydride and catalytic amount of NaHSO_4_-SiO_2_ under solvent-free conditions (Scheme 1).

Initially we attempted the acylation reaction of benzaldehyde with acetic anhydride in the absence of NaHSO_4_-SiO_2_. The reaction was sluggish and no corresponding 1,1-diacetate was formed even after 24 h. However in the presence of NaHSO_4_-SiO_2_ (25%/wt) the reaction progressed smoothly with benzaldehyde at room temperature to afforded the corresponding 1,1-diacetate with excellent yield. Therefore, we employed the above conditions for the conversion of various aldehydes to the corresponding acylals under solvent-free conditions (Table 1).

**Table 1:** **Formation of acylals using NaHSO**_**4**_-**SiO**_**2**_** under solvent**-**free conditions at rt**^**a**^

**Entry**	**Substrate**	**Product**	**Time (min)**	**Yield (%)**^**b**^	**M.p °C (Lit)**
1			15	94	45-46
					(44–45) [27]
2			20	88	82-84
					(82–83) [28]
3			20	90	83-85
					(84) [29]
4			15	91	81-83
					(81–82) [30]
5			15	89	69-71
					(68–70) [28]
6			25	84	64-65
					(65–66) [28]
7			20	90	86-88
					(88) [27]
8			15	94	124-126
					(125) [29]
9			20	85	50-52
					(52–53) [28]
10			15	87	Oil [27]

^a^Reaction conditions: benzaldehyde (1 mmol), Ac_2_O (4 mmol) and NaHSO_4_-SiO_2_ (25%/wt) were stirred at RT under solvent-free conditions,^b^ isolated yields.

**Scheme 2:**
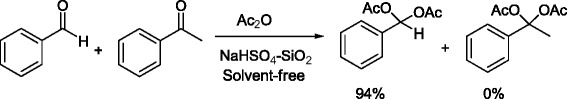
Chemoselective synthesis acylal from aldehyde.

The results listed in Table 1 show that both aromatic and aliphatic aldehydes reacts smoothly with acetic anhydride to afford the corresponding 1,1-diacetates in good to excellent yields. Aromatic aldehydes possessing electron-withdrawing substituents, halogens and electron-releasing substituents on the aromatic ring afforded the corresponding acylals in Excellent yields and in short reaction times. Nitro substituted aldehydes are also produced good yields, but the powerful electron-releasing substituent OMe slightly decreased the yield and increased the reaction time. In order to show the high selectivity of the method, we studied competitive reactions for the acylation of aldehydes in the presence of ketones using silica supported sodium hydrogen sulphate as a catalyst. Using this catalytic system, the highly selective conversion of an aldehyde in the presence of ketone was observed (Scheme 2).

**Table 2:** **The synthesis of acylal from benzaldehyde and acetic anhydride in the presence of recycled NaHSO**_**4**_-**SiO**_**2**_

**Entry**	**Cycle**	**Time(min)**	**Yield(%**)^a^
1	1^st^ use	15	94
2	2^nd^ use	20	89
3	3^rd^ use	30	77

^a^Isolated yield.

The recycling of the catalyst is one of the most advantages of our method. For the reaction of benzaldehyde with acetic anhydride good yield was observed when NaHSO_4_-SiO_2_ was reused even after three times recycling (Table 2).

## Conclusion

In conclusion, NaHSO_4_-SiO_2_ is a chemoselective and highly efficient catalyst for acylal formation from aldehydes. The advantages of this methodology over the reported methods is the availability of the starting materials, simplicity of acylation procedure, a clean work-up, a short reaction time, and high yields. In addition, this reagent acts as a heterogeneous catalyst that could be removed from the reaction mixture by simple filtration and compliance with the green chemistry protocols.

## Experimental section

All ^1^ H NMR spectra were recorded on 400 MHz Varian FT-NMR spectrometers. All chemical shifts are given as δ value with reference to Tetra methyl silane (TMS) as an internal standard. Melting points were taken in open capillaries. The IR spectra were recorded on a PerkinElmer 257 spectrometer using KBr discs. Products were purified by flash chromatography on 100–200 mesh silica gel. The chemicals and solvents were purchased from commercial suppliers either from Aldrich, Spectrochem and they were used without purification prior to use. The obtained products were characterized from their spectral (^1^ H-NMR, IR) and comparison to authentic samples.

### Preparation of silica supported sodium hydrogen sulphate

To a solution of 4.14 g (0.03 mol) of NaHSO_4_.H_2_O in 20 mL of water in a 100 mL beaker containing a stir bar was added 10 g of SiO_2_ (column chromatographic grade, 230–400 mesh). The mixture was stirred for 15 min and then gently heated on a hot plate, with intermittent swirling, until a free-flowing white solid was obtained. The catalyst was further dried by placing the beaker in an oven maintained at 120 °C for at least 48 h prior to use.

### General experimental procedure

A mixture of aldehyde (2 mmol), freshly distilled Ac_2_O (8 mmol) and NaHSO_4_-SiO_2_ (25%/wt) was stirred at room temperature and the progress of the reaction was monitored by TLC Hexane: EtOAc (9:1) after completion of the reaction, the reaction mixture was treated by dilution with EtOAc and the catalyst was removed by filtration. Obtained filtrate was washed with saturated NaHCO_3_ solution and water and then dried over Na_2_SO_4_. The solvent was evaporated under reduced pressure to get the crude product was purified by column chromatography to give pure acylal compound.

### Selected spectral data of the products

**Phenylmethylene diacetate** (Table 1, Entry-1)

^1^ H NMR (CDCl_3_): δ7.63(s, 1 H), 7.53-7.50 (m, 2 H), 7.40-7.36 (m, 3 H), 2.11 (s, 6 H); IR (KBr, cm^-1^): 3068, 1756, 1504, 1440, 1010; Anal. Calcd. For C_11_H_12_O_4_: C, 63.45; H, 5.81. Found: C, 63.71; H, 5.70.

(**4**-**chlorophenyl**) **methylene diacetate** (Table 1, Entry-2)

^1^ H NMR (CDCl_3_): δ7.63 (s, 1 H), 7.46 (d, *J* = 8.4 Hz, 2 H), 7.38 (d, *J* = 8.4 Hz, 2 H), 2.12 (s, 6 H); IR (KBr, cm^-1^): 3019, 2924, 1769, 1745, 1492, 1373, 1241, 1070, 1006; Anal. Calcd. For C_11_H_11_ClO_4_; C, 54.45; H, 4.57. Found: C, 54.36; H, 4.68.

(**4**-**methoxyphenyl**) **methylene diacetate** (Table 1, Entry-6)

^1^ H NMR (CDCl_3_): δ7.62 (s, 1 H), 7.45 (d, *J* = 8.8 Hz, 2 H), 6.92 (d, *J* = 8.8 Hz, 2 H), 3.82 (s, 3 H), 2.11 (s, 6 H); IR (KBr, cm^-1^): 3014, 2937, 1749, 1618, 1378, 1244, 1207, 1018, 936; Anal. Calcd. For C_12_H_14_O_5_; C, 60.50; H, 5.92. Found: C, 60.98; H, 5.66.

## Competing interests

The authors declare that they have no competing interests.

## Authors' contributions

RKK the main author completed this work under the guidance of VVSP as the corresponding author, and SRB as the coauthor. All authors read and approved the final manuscript.
